# FINDRISC in Latin America: a systematic review of diagnosis and prognosis models

**DOI:** 10.1136/bmjdrc-2019-001169

**Published:** 2020-04-22

**Authors:** Rodrigo M Carrillo-Larco, Diego J Aparcana-Granda, Jhonatan R Mejia, Antonio Bernabé-Ortiz

**Affiliations:** 1 Department of Epidemiology and Biostatistics, School of Public Health, Imperial College London, London, UK; 2 CRONICAS Centre of Excellence in Chronic Diseases, Universidad Peruana Cayetano Heredia, Lima, Peru; 3 Instituto de Investigación, Universidad Católica Los Ángeles de Chimbote, Chimbote, Peru; 4 Facultad de Medicina Humana, Universidad Nacional del Centro del Perú, Huancayo, Peru; 5 Universidad Científica del Sur, Lima, Peru

**Keywords:** type 2 diabetes mellitus, prognostic models, diagnostic models, low- and middle-income countries, FINDRISC

## Abstract

This review aimed to assess whether the FINDRISC, a risk score for type 2 diabetes mellitus (T2DM), has been externally validated in Latin America and the Caribbean (LAC). We conducted a systematic review following the CHARMS (CHecklist for critical Appraisal and data extraction for systematic Reviews of prediction Modelling Studies) framework. Reports were included if they validated or re-estimated the FINDRISC in population-based samples, health facilities or administrative data. Reports were excluded if they only studied patients or at-risk individuals. The search was conducted in Medline, Embase, Global Health, Scopus and LILACS. Risk of bias was assessed with the PROBAST (Prediction model Risk of Bias ASsessment Tool) tool. From 1582 titles and abstracts, 4 (n=7502) reports were included for qualitative summary. All reports were from South America; there were slightly more women, and the mean age ranged from 29.5 to 49.7 years. Undiagnosed T2DM prevalence ranged from 2.6% to 5.1%. None of the studies conducted an independent external validation of the FINDRISC; conversely, they used the same (or very similar) predictors to fit a new model. None of the studies reported calibration metrics. The area under the receiver operating curve was consistently above 65.0%. All studies had high risk of bias. There has not been any external validation of the FINDRISC model in LAC. Selected reports re-estimated the FINDRISC, although they have several methodological limitations. There is a need for big data to develop—or improve—T2DM diagnostic and prognostic models in LAC. This could benefit T2DM screening and early diagnosis.

## Introduction

With an increasing load in terms of prevalence,^1^ disability and mortality,^2–4^ as well as economic burden,^5^ type 2 diabetes mellitus (T2DM) is a global threat to population health and health systems, especially in low-income and middle-income countries.^1–5^ Although universal health coverage should secure treatment for all patients, this goal may not be realistic where there is not universal screening and where resources are limited to identify all at-risk populations. Therefore, inexpensive yet reliable screening tools could be useful to identify T2DM cases or high-risk people. Risk scores, both diagnostic and prognostic, help identify people at high risk of having or developing T2DM. This way, these people could undergo further diagnostic tests, primary prevention or receive pharmacological treatment as needed. Nonetheless, risk scores need to be tested, and possibly adapted (ie, recalibrated), to produce accurate estimates to inform health decisions. Several T2DM risk scores have been developed,^6–9^ although very few for Latin America and the Caribbean (LAC), where those available exhibit major limitations hindering their implementation across countries or their endorsement by policies or guidelines.^10^ A well-known T2DM risk score is the FINDRISC,^11^ which is also acknowledged by the Latin American diabetes guidelines as an available diabetes screening tool;^12^ yet it is unknown if this model has been appropriately adapted in LAC. Consequently, we aimed to describe and assess if external validations of the FINDRISC model in LAC were conducted following adequate methods.^13 14^ We will complement the available evidence about T2DM risk scores in LAC^10^ and inform regional guidelines,^12^ while also pinpointing research priorities and policies for T2DM screening and early diagnosis through risk stratification.^15 16^

## Methods

### Protocol

This systematic review and critical appraisal of the scientific literature adheres to the Preferred Reporting Items for Systematic Reviews and Meta-Analyses guidelines (online supplementary material). We followed the CHARMS (CHecklist for critical Appraisal and data extraction for systematic Reviews of prediction Modelling Studies) methodology to formulate the review framework, research question and strategy (table 1).^17 18^

10.1136/bmjdrc-2019-001169.supp1 Supplementary data

**Table 1:** CHARMS criteria to define research question and strategy

Concept	Criteria
Prognostic or diagnostic?	The review focused on FINDRISC regardless if it was studied as a diagnostic or prognostic model for T2DM.
Scope	To inform physicians, researchers and the general population whether they are likely to have T2DM (ie, diagnostic) or will be likely to have T2DM (ie, prognostic). FINDRISC models could be used for research, screening and treatment allocation in primary prevention.
Type of prediction modeling studies	Diagnostic/prognostic models with external validation. Diagnostic/prognostic models without external validation. Diagnostic/prognostic model validation.
Target population to whom the prediction model applies	General adult population in LAC.
Outcome to be predicted	T2DM.
Time span of prediction	Prognostic models will not be included/excluded based on prediction time; that is, it could be short term (eg, next 2.5 years) or long term (eg, next 10 years).
Intended moment of using the model	FINDRISC models to be used in asymptomatic adults of LAC to assess their probability to have T2DM (ie, diagnostic) or their probability to develop T2DM in a predefined period (ie, prognostic).

Based on the CHARMS checklist.^18^

CHARMS, CHecklist for critical Appraisal and data extraction for systematic Reviews of prediction Modelling Studies; LAC, Latin America and the Caribbean; T2DM, type 2 diabetes mellitus.

### Information sources

The search strategy was conducted in five search engines: Embase, Medline and Global Health through OVID, and also in Scopus and LILACS. The search was conducted on September 28, 2019. The search terms are available in the online supplementary material.

### Eligibility criteria

We sought FINDRISC models aiming to inform about the current (diagnostic) or future (prognostic) risk of T2DM in LAC populations. Selected original reports could have developed a new model using the same (or very similar) predictors as in the original FINDRISC^11^; similarly, they could have performed an independent external validation in LAC populations. The outcome of the diagnostic or prognostic FINDRISC models was T2DM. The outcome should have been ascertained with at least one biomarker (eg, fasting glucose, hemoglobin A1c (HbA1c) or oral glucose tolerance test). Thus, we did not include studies where the outcome relied entirely on self-reported diagnosis. We focused on adult men and women.

### Study selection

Original scientific reports were excluded if the study population only included people with a disease (eg, patients with hypertension) or based on a risk factor (eg, smokers). Similarly, studies with LAC populations in countries outside LAC were excluded (eg, Hispanics in the USA). Conversely, reports were included if they followed a probabilistic population-based sampling approach, were based on primary care settings, or were based on health or claims registries or administrative data. The original work should have focused on the FINDRISC model, regardless of whether they developed an identical new model, a very similar model, or independently externally validated the FINDRISC model. Studies were included if they followed a cross-sectional or prospective observational design.

### Data collation process

We used EndNote and Rayyan^19^ to remove duplicates from the search. First, we used Rayyan^19^ to screen titles and abstracts, which were screened by two reviewers independently (pairwise combinations between RMC-L and DJA-G or JRM); discrepancies were solved by consensus. Then, two reviewers independently (pairwise combinations between RMC-L and DJA-G or JRM) studied the full text of those reports selected in the screening phase; discrepancies were solved by consensus. If consensus could not be reached, discrepancies were solved by a third party (AB-O).

The authors developed a data extraction form based on international guidelines for systematic reviews of prognosis models^17 18^ and on a previous systematic review on the subject.^10^ The data extraction form was not modified during data collation. Information was extracted as presented in the original reports by two reviewers independently (pairwise combinations between RMC-L and DJA-G or JRM); discrepancies were solved by consensus.

### Risk of bias of individual studies

Using the PROBAST (Prediction model Risk of Bias ASsessment Tool) tool for risk of bias assessment of prognosis models,^20 21^ two reviewers (DJA-G and JRM) independently assessed the risk of bias of the selected reports. If there were any discrepancies, these were solved by consensus or by a third party (RMC-L).

### Synthesis of results

Because of the limited numbers of results and the great heterogeneity among them, only a qualitative synthesis was conducted.

## Results

### Reports selection

**Figure 1:**
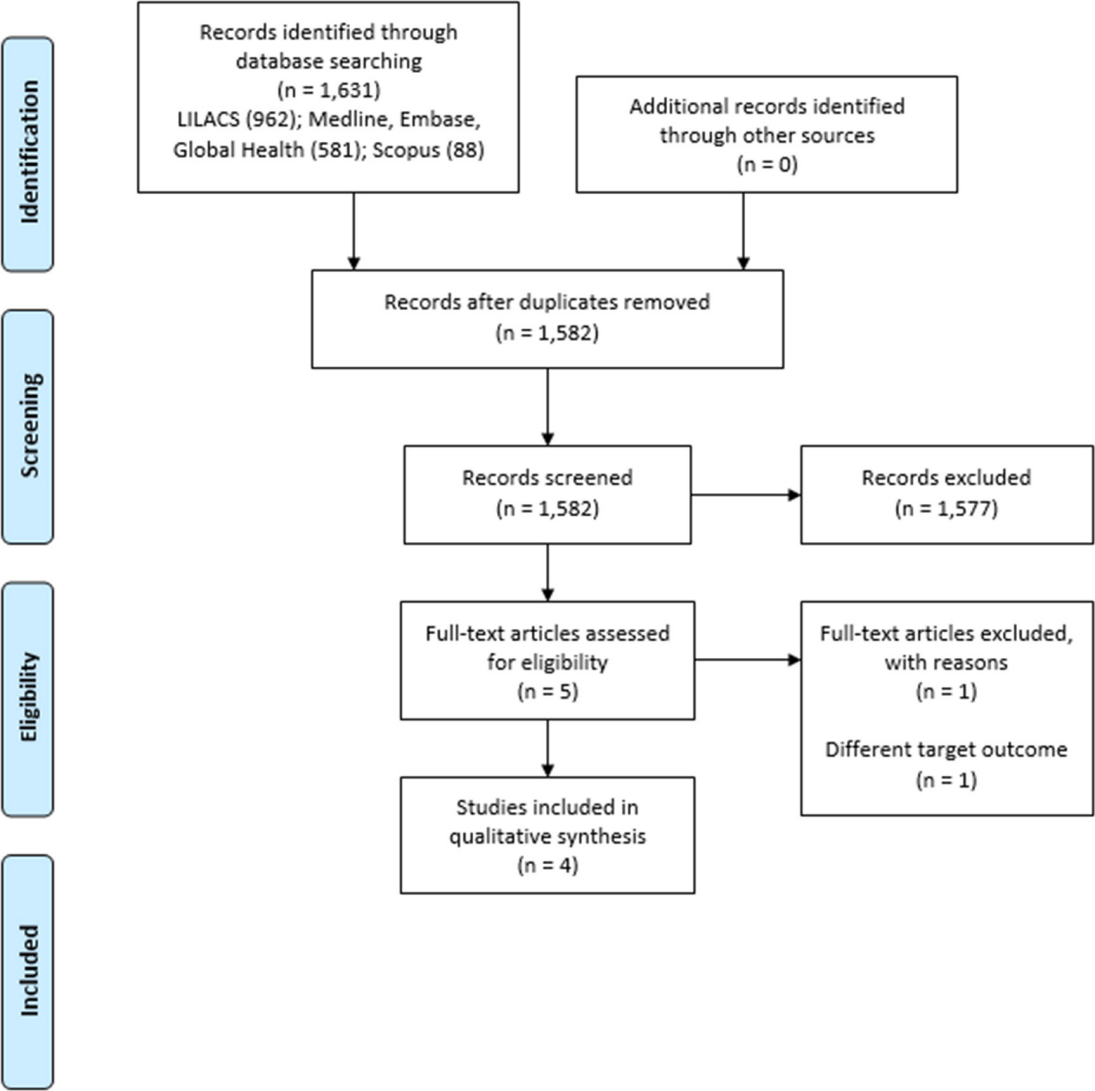
Flow chart of the study selection process.

The screening process included 1582 titles and abstracts, of which 1577 were excluded. Therefore, five reports were studied in full text. One report was excluded because they assessed a different outcome.^22^ Finally, four reports (n=7502) were included in the qualitative review (figure 1).^23–26^

### General characteristics

All the reports were from South America: one from Peru,^24^ one from Venezuela,^26^ and two from Colombia.^23 25^ Bernabe-Ortiz *et al*^24^ as well as Nieto-Martínez *et al*^26^ studied population-based samples while Gomez-Arbelaez *et al*^25^ and Barengo *et al*^23^ analyzed data from health centers (online supplementary material).

The largest sample size was studied by Nieto-Martínez *et al* (n=3061); this was also a national representative sample.^26^ The smallest sample was studied by Gomez-Arbelaez and colleagues (n=772).^25^ The studied samples tended to include slightly more women than men, except for one report (58.3% men).^25^ The mean age ranged from 29.5 to 49.7 years (table 2, online supplementary material).^24 25^

**Table 2:** General characteristics

Study	Outcome prevalence (%)	Mean age (years)	Men (%)	Outcome details	Baseline sample size	Outcome events (n)	Outcome events per candidate predictors
Gomez-Arbelaez *et al*^25^	2.59	58.34	29.53	The diagnosis of type 2 diabetes mellitus was established when fasting plasma glucose ≥126 mg/dL, OGTT ≥200 mg/dL and/or HbA1c ≥6.5%.	772	20	
Bernabe-Ortiz *et al*^24^ FINDRISC Simplified, FINDRISC and LA-FINDRISC	4.70	48.20	49.70	Individuals who were not aware of having type 2 diabetes mellitus and had fasting glucose ≥126 mg/dL (≥7.0 mmol/L) or 2-hour plasma glucose ≥200 mg/dL (≥11.1 mmol/L).	1609	71	5.92
Nieto-Martinez *et al*^26^ LA-FINDRISC and FINDRISC	3.30	39.90	46.97	Diabetes was defined if the fasting plasma glucose was ≥126 mg/dL or if the 2-hour OGTT glucose was ≥200 mg/dL.	3061	101	
Barengo *et al*^23^ ColDRISC and FINDRISC	5.10	47.20	38.00	Individuals who had fasting plasma glucose ≥126 mg/dL or 2-hour plasma glucose ≥200 mg/dL were classified as having T2DM.	2060	105	11.67

HbA1c, hemoglobin A1c; OGTT, oral glucose tolerance test.

Across reports, T2DM was ascertained with a combination of fasting plasma glucose ≥126 mg/dL, HbA1c ≥6.5% or 2-hour plasma glucose ≥200 mg/dL (table 2, online supplementary material).^23–26^ Undiagnosed T2DM prevalence was largest in the report by Barengo *et al* (5.10%; n=105),^23^ followed by the study in Peru (4.70%; n=71),^24^ the report from Venezuela (3.30%; n=101)^26^ and the work by Gomez-Arbelaez *et al*^25^ (2.59%; n=20) (table 2, online supplementary material).

### Predictors and modeling

None of the studies conducted an independent external validation of the FINDRISC model. Conversely, they used the same (or very similar) predictors to fit a new model.^23–26^ In so doing, they all produced new coefficients and baseline risks.^23–26^ As in the original FINDRISC,^11^ numeric variables were categorized. The modeling strategy was consistently logistic regression, and complete-case analyses were conducted (online supplementary material).

Two authors developed new risk scores.^23 24^ Barengo *et al*^23^ started with nine candidate predictors to develop a Colombian version of the FINDRISC with six predictors; predictor selection was based on univariate analysis (online supplementary material). Bernabe-Ortiz *et al*^24^ developed a simplified version of the FINDRISC including 5 predictors, yet there were 12 candidate predictors selected through stepwise backward elimination (online supplementary material).

### Model performance

None of the studies reported any calibration metrics (online supplementary material).^23–26^ Conversely, they all focused on discrimination (area under the receiver operating curve) and other classification metrics, including sensitivity, specificity, and positive and negative predictive values. The area under the receiver operating curve was consistently above 65.0% (table 3, online supplementary material).

**Table 3:** Performance metrics

First author, and assessed model	Discrimination (%)	Classification measures
Gomez-Arbelaez *et al*^25^	74.77 (95% CI 57.22 to 92.32) (men) and 71.75 (95% CI 58.68 to 84.81) (women)	At a cut-off of ≥14. Men: sensitivity=66.7; specificity=75.2; positive predictive value=6.8; negative predictive value=98.8; Youden’s index=0.419. Women: sensitivity=71.4; specificity=62.6; positive predictive value=4.8; negative predictive value=98.8; Youden’s index=0.340.
Bernabe-Ortiz *et al*,^24^ FINDRISC Simplified	71.10	At a cut-off of 3: sensitivity=0.859; specificity=0.467; positive predictive value=0.074; negative predictive value=0.985; likelihood ratio positive=1.6; likelihood ratio negative=0.3; diagnostic OR=5.3.
Bernabe-Ortiz *et al*,^24^ FINDRISC	69.00	At a cut-off of 11: sensitivity=0.690; specificity=0.668; positive predictive value=0.094; negative predictive value=0.978; likelihood ratio positive=2.1; likelihood ratio negative=0.5; diagnostic OR=4.5.
Bernabe-Ortiz *et al*,^24^ LA-FINDRISC	68.00	At a cut-off of 10: sensitivity=0.704; specificity=0.591; positive predictive value=0.079; negative predictive value=0.970; likelihood ratio positive=1.7; likelihood ratio negative=0.5; diagnostic OR=3.4.
Nieto-Martinez *et al*,^26^ LA-FINDRISC	72.2 (95% CI 66.8 to 77.5) (men) and 72.40 (95% CI 63.9 to 81.0) (women)	For men at a cut-off of 9: sensitivity=72.2; specificity=62.2; positive likelihood ratio=1.91. For women at a cut-off of 10: sensitivity=71.4; specificity=65.4; positive likelihood ratio=2.06.
Nieto-Martinez *et al*,^26^ FINDRISC	72.90 (95% CI 67.6 to 78.1) (men) and 73.20 (95% CI 64.8 to 81.6) (women)	
Barengo *et al*,^23^ ColDRISC	74 (95% CI 70 to 79)	At a cut-off of 4: sensitivity=0.73; specificity=0.67; positive predictive value=0.106; negative predictive value=0.979.
Barengo *et al*,^23^ modified FINDRISC	73 (95% CI 69 to 78)	At a cut-off of 10: sensitivity=0.72; specificity=0.60; positive predictive value=0.084; negative predictive value=0.984.

### Risk of bias

All studies exhibited high risk of bias mainly due to limitations in the analytical approach, for example limited number of outcome events. In this line, a complete-case analysis was consistently preferred versus multiple imputation. Most importantly, calibration metrics were consistently not reported. On the other hand, participants, predictors, and outcome criteria showed low risk of bias. There was low applicability concern (table 4, online supplementary material).

**Table 4:** Risk of bias assessment of individual diagnostic/prediction models (PROBAST)^20 21^

First author and assessed model	RoB				Applicability			Overall	
	Participants	Predictors	Outcome	Analysis	Participants	Predictors	Outcome	RoB	Applicability
Barengo *et al*,^23^ ColDRISC	+	+	+	−	+	+	+	−	+
Barengo *et al*,^23^ modified FINDRISC	+	+	+	−	+	+	+	−	+
Bernabe-Ortiz *et al*,^24^ FINDRISC Simplified	+	+	+	−	+	+	+	−	+
Bernabe-Ortiz *et al*,^24^ FINDRISC	+	+	+	−	+	+	+	−	+
Bernabe-Ortiz *et al*,^24^ LA-FINDRISC	+	+	+	−	+	+	+	−	+
Gomez-Arbelaez *et al*^25^	+	+	+	−	+	+	+	−	+
Nieto-Martínez *et al*,^26^ LA-FINDRISC	+	+	+	−	+	+	+	−	+
Nieto-Martínez *et al*,^26^ FINDRISC	+	+	+	−	+	+	+	−	+

+ indicates low RoB/low concern regarding applicability; − indicates high RoB/high concern regarding applicability.

PROBAST, Prediction model Risk of Bias ASsessment Tool; RoB, risk of bias.

## Discussion

### Main findings

This review did not find any independent external validations of the original FINDRISC model in LAC, as the four reports herein described re-estimated the FINDRISC model; in other words, authors computed new coefficients and baseline risks instead of using the original ones to test the model performance in a new population with subsequent recalibration if needed. While the analyzed reports exhibited methodological limitations, including reduced number of outcome events and not reporting calibration metrics, they showed acceptable discrimination performance. In LAC, risk prediction research needs to be improved to generate reliable tools for risk stratification, which could offer a cost-effective approach in the cascade to identify new and future T2DM cases.^27^

### Limitations of the review

Although we followed a comprehensive methodology, there are still limitations to be acknowledged. First, we did not search gray literature; however, we would not expect results from these sources, if any, to substantially change the main findings or conclusions of this review. Second, the focus of this work was on LAC; whether our findings apply to other world regions mostly hosting low-income and middle-income countries deserves further verification.

### Limitations of the selected reports

We have previously pinpointed several methodological limitations of T2DM risk scores in LAC,^10^ and these would also apply to those herein studied. Although there is literature addressing good methods for development and validation of risk scores,^13 14^ the most recurrent pitfall herein identified is the limited number of outcome events, which may allow including few predictors or could lead to overfitting of the prediction model. We understand that (big) data with enough outcome events may be scarce in LAC; thus, we value and acknowledge the available research. Recently, methods have been developed to define sample size for risk prediction models with binary outcomes.^28^ Where possible, researchers could adhere to these standards.

We strongly believe there is a great need to look for (big) data, for example, national surveys (eg, WHO STEPwise approach to Surveillance (STEPS) or Demographic and Health Surveys (DHS)). These surveys are available in many countries, and pooling them, following adequate techniques, could generate a rich database to develop a T2DM risk score for LAC. Finally, it is also worrying that none of the studies reported calibration metrics such as calibration slope, calibration in the large or calibration plots.^13 14^ Calibration refers to the agreement between observed and predicted events.^13 14^ Therefore, it provides information to understand whether the model is underestimating (observed > predicted) or overestimating (observed < predicted) the outcome. As risk prediction research further penetrates in LAC, standard and sound methods should be adopted and reported appropriately; thereby, robust tools will be available to be incorporated in health policies and guidelines.^13 14 29^

The summarized reports also provided metrics usually available for diagnostic tools, including positive/negative predictive values and positive/negative likelihood ratios. Of these additional metrics, the negative predictive value was the largest, consistently above 97%. This refers that a subject with a negative test is in fact disease-free. In other words, of 100 people who take the test and have a negative result, over 97 of them would not have the disease. This metric depends on the prevalence in the underlying population; that is, this is not an intrinsic property of the model. Thus, this metric would not be useful in generalizing the accuracy of the test across populations with different prevalences. Nonetheless, this could suggest that for people with a very low score or a score below a established threshold, further tests are not needed because they are most likely not to have diabetes.

### Clinical and public health relevance

The American^30^ and Canadian^31^ T2DM guidelines include risk scores to identify people who would need further laboratory tests to confirm T2DM;^30 31^ these documents suggest specific risk scores such as the Canadian Diabetes Risk Assessment Questionnaire.^31^ The LAC guidelines, on the other hand, support the use of risk scores for screening purposes, without advocating for any tool in particular, although they acknowledge the FINDRISC as a relevant and useful tool.^12^ Probably the LAC guidelines do not support a risk score in particular due to the dearth of tools and the limitations of the few available ones.^10^ Following the example of the US and Canadian guidelines, LAC T2DM institutions should support and foster the development of a strong T2DM risk score, which could benefit from national survey data or large pooling data endeavors.

Whether risk scores are the best method to screen for diabetes is yet to be known. Other alternatives include massive screening with blood tests (eg, random glucose or HbA1c) or screening based on single risk factors (eg, people with severe obesity). The first alternative may not be feasible in low-income and middle-income countries or rural settings, where costs and scarce laboratory facilities may preclude this option. Screening on single risk factors may not be sensible enough, hence the need for risk scores to combine several predictors to compute a more comprehensive probability. Also, there is evidence suggesting that screening with a risk stratification tool, such as a risk score, is a cost-effective approach.^27^ While other screening methods are being developed and proven better than risk scores, risks scores need to be improved to provide accurate results that can inform public health (eg, number of people at risk in need of tests) and clinical medicine (eg, when to start counseling or treatment). The diabetes guidelines for Latin America do not explicitly recommend using the FINDRISC, yet they signal the FINDRISC as a relevant screening tool.^12^ In Colombia, on the other hand, clinical guidelines do recommend the FINDRISC.^32^ Assessing which guidelines recommend the FINDRISC, or other risk scores, is beyond the scope of this work. However, given the limitations herein pinpointed as well as by a previous systematic review on the subject,^10^ we recommend cautious use of available tools, particularly if they are being used in populations different from those used in developing the model.

## Conclusions

There has not been an external validation of the FINDRISC model in LAC, where several re-estimations of this model have been conducted. The available research has benefitted from studies with limited coverage, for example, small cross-sectional studies. This calls to strengthen the use of (big) data or national surveys across LAC to develop—or improve—T2DM diagnostic and prognostic risk scores. This could have large positive impact on T2DM screening and early diagnosis in LAC. Overall, the discrimination accuracy of the FINDRISC in LAC seems adequate, although no evidence is available on calibration metrics.
