# Supplementary material for: FINDRISC in Latin America: a systematic review of diagnosis and prognosis models
Source: BMJ Open Diabetes Res Care. 2020 Apr 22;8(1):e001169. doi: 10.1136/bmjdrc-2019-001169 (PMC7202717; doi:10.1136/bmjdrc-2019-001169)
Supplement: Supplementary data [file bmjdrc-2019-001169supp001.pdf]

Supplementary Material

FINDRISC in Latin America: A systematic review of diagnosis and prognosis models

Contents

Search terms 2
- Embase, Medline and Global Health (OVID) 2
- SCOPUS 3
- LILACS 4

Data extraction form (by chapters) 5
- Source of data and participants 5
- Outcome 7
- Candidate predictors 8
- Sample size and missing data 10
- Model development 11
- Model performance 12
- Results 13
- Discussion 14

Risk of Bias (RoB) 15

Applicability 18

PRISMA Checklist 19

Carrillo-Larco RM, *et al. BMJ Open Diab Res Care* 2020; 8:e001169. doi: 10.1136/bmjdrc-2019-001169

Search terms

Embase, Medline and Global Health (OVID)

01	type 2 diabetes.mp.
02	T2D*.mp.
03	exp Diabetes Mellitus/
04	exp Diabetes Mellitus, Type 2/
05	pre-diabetes.mp.
06	pre-diabetic.mp.
07	prediabetic state.mp.
08	diabetes.mp.
09	("type 2" or type two or type ii or type II).mp.
10	08 and 09
11	01 or 02 or 03 or 04 or 07 or 10
12	risk assessment.mp.
13	risk functions.mp.
14	Risk Assessment/mt
15	risk equation$.mp.
16	risk chart?.mp.
17	(risk adj3 tool$).mp.
18	risk assessment function?.mp.
19	risk assessor.mp.
20	risk appraisal$.mp.
21	risk calculation$.mp.
22	risk calculator$.mp.
23	risk factor$ calculator$.mp.
24	risk factor$ calculation$.mp.
25	risk engine$.mp.
26	risk equation$.mp.
27	risk table$.mp.
28	risk threshold$.mp.
29	risk disc?.mp.
30	risk disk?.mp.
31	risk scoring method?.mp.
32	scoring scheme?.mp.
33	risk scoring system?.mp.
34	risk scal$.mp.
35	risk prediction?.mp.
36	risk algorith$.mp.
37	prediction model$.mp.
38	predictive instrument?.mp.
39	project$ risk?.mp.
40	predictive model?.mp.
41	scoring method$.mp.
42	(prediction$ adj3 method$).mp.
43	exp Risk Assessment/
44	(risk? adj1 assess$).mp.
45	screening.mp.

Carrillo-Larco RM, *et al. BMJ Open Diab Res Care* 2020; 8:e001169. doi: 10.1136/bmjdrc-2019-001169

46	diagnostic test.mp.
47	12 or … 46
48	Finnish Diabetes Risk Score.mp.
49	FINDRISC.mp.
50	Latin-American FINDRISC.mp.
51	LA-FINDRISC.mp.
52	48 or 49 or 50 or 51
53	47 or 52
54	("Antigua and Barbuda" or "Argentina" or "Bahamas" or "Barbados" or "Belize" or "Bolivia" or "Brazil" or "United States Virgin Islands" or "British Virgin Islands" or "Chile" or "Colombia" or "Costa Rica" or "Cuba" or "Dominica" or "Dominican Republic" or "Ecuador" or "El Salvador" or "Grenada" or "Guatemala" or "Guyana" or "Haiti" or "Honduras" or "Jamaica" or "Mexico" or "Nicaragua" or "Panama" or "Paraguay" or "Peru" or "Puerto Rico" or "Saint Kitts and Nevis" or "Saint Lucia" or "Saint Vincent and the Grenadines" or "Suriname" or "Trinidad and Tobago" or "West Indies" or "Uruguay" or "Venezuela" or "Latin America" or latin amer$ or "South America" or south amer$ or "Central America" or central amer$ or "Caribbean Region").mp.
55	11 and 53 and 54
56	exp animals/ not humans.sh.
57	55 not 56
58	Remove duplicates from 57

SCOPUS

(TITLE-ABS-KEY(diabetes mellitus) OR TITLE-ABS-KEY (Diabetes Mellitus, Type 2) OR TITLE-ABS-KEY(diabetes type 2) OR TITLE-ABS-KEY (type 2 diabetes) OR TITLE-ABS-KEY(pre-diabetes) OR TITLE-ABS-KEY(pre-diabetic) OR TITLE-ABS-KEY(prediabetic state)) AND (TITLE-ABS-KEY(Risk Assessment) OR TITLE-ABS-KEY(risk? adj1 assess*) OR TITLE-ABS-KEY(risk function) OR TITLE-ABS-KEY(Risk Assessment) OR TITLE-ABS-KEY(risk functions) OR TITLE-ABS-KEY(risk equation*) OR TITLE-ABS-KEY(risk chart?) OR TITLE-ABS-KEY(risk adj3 tool*) OR TITLE-ABS-KEY(risk assessment function?) OR TITLE-ABS-KEY(risk assessor) OR TITLE-ABS-KEY(risk appraisal*) OR TITLE-ABS-KEY(risk calculation*) OR TITLE-ABS-KEY(risk calculator*) OR TITLE-ABS-KEY(risk factor* calculator*) OR TITLE-ABS-KEY(risk factor* calculation*) OR TITLE-ABS-KEY(risk engine*) OR TITLE-ABS-KEY(risk equation*) OR TITLE-ABS-KEY(risk table*) OR TITLE-ABS-KEY(risk threshold*) OR TITLE-ABS-KEY(risk disc?) OR TITLE-ABS-KEY(risk disk?) OR TITLE-ABS-KEY(risk scoring method?) OR TITLE-ABS-KEY(scoring scheme?) OR TITLE-ABS-KEY(risk scoring system?) OR TITLE-ABS-KEY(risk prediction?) OR TITLE-ABS-KEY(risk algorith*) OR TITLE-ABS-KEY(prediction model*) OR TITLE-ABS-KEY(predictive instrument?) OR TITLE-ABS-KEY(project* risk?) OR TITLE-ABS-KEY(predictive model?) OR TITLE-ABS-KEY(scoring method*) OR TITLE-ABS-KEY(prediction* adj3 method*) OR TITLE-ABS-KEY(screening) OR TITLE-ABS-KEY(risk scal*) OR TITLE-ABS-KEY(diagnostic test) OR TITLE-ABS-KEY(Finnish Diabetes Risk Score) OR TITLE-ABS-KEY(FINDRISC) OR TITLE-ABS-KEY(Latin-American FINDRISC) OR TITLE-ABS-KEY(LA-FINDRISC)) AND TITLE-ABS-KEY("Antigua and Barbuda" OR "Argentina" OR "Bahamas" OR "Barbados" OR "Belize" OR "Bolivia" OR "Brazil" OR "United States Virgin Islands" OR "British Virgin Islands" OR "Chile" OR "Colombia" OR "Costa Rica" OR "Cuba" OR "Dominica" OR "Dominican Republic" OR "Ecuador" OR "El Salvador" OR "Grenada" OR "Guatemala" OR "Guyana" OR "Haiti" OR "Honduras" OR "Jamaica" OR "Mexico" OR "Nicaragua" OR "Panama" OR "Paraguay" OR "Peru" OR "Puerto Rico" OR "Saint Kitts and Nevis" OR "Saint Lucia" OR "Saint Vincent and the Grenadines" OR "Suriname"

OR "Trinidad and Tobago" OR "West Indies" OR "Uruguay" OR "Venezuela" OR "Latin America" OR latin amer* OR "South America" OR south amer* OR "Central America" OR central amer* OR "Caribbean Region") AND NOT DBCOLL(medl)

LILACS

((diabetes mellitus) OR (diabetes tipo 2) OR (diabetes mellitus tipo 2) OR (diabetes) OR (pre-diabetes) OR (pre-diabetic$) OR (estado prediabetico)) AND ((funcion de riesgo) OR (evaluacion del riesgo) OR (funcion$ de riesgo) OR (ecuacion de riesgo) OR (tabla de riesgos) OR (herramienta de ajuste de riesgo) OR (funcion de evaluación de riesgo) OR (asesor de riesgo) OR (calculo de riesgo) OR (calculadora de riesgo) OR (motor de riesgo) OR (umbral de riesgo) OR (metodo de calificacion de riesgo) OR (esquema de puntuacion) OR (sistema de puntuacion de riesgo) OR (prediccion de riesgo) OR (algoritmo de riesgo) OR (modelo predictivo) OR (prediccion de riesgo) OR (modelo de prediccion) OR (instrumento predictivo) OR (proyecto$ riesgo) OR (tamizaje) OR (escala de riesgo) OR (Finnish Diabetes Risk Score) OR (FINDRISC) OR (Latin-American FINDRISC) OR (LA-FINDRISC)) AND (("Antigua y Barbuda") OR ("Argentina") OR ("Aruba") OR ("Bahamas") OR ("Barbados") OR ("Belice") OR ("Bolivia") OR ("Brasil") OR ("Islas Vírgenes de los Estados Unidos") OR ("Islas Vírgenes Británicas") OR ("Islas Caimán") OR ("Chile") OR ("Colombia") OR ("Costa Rica") OR ("Cuba") OR ("Curazao") OR ("Dominica") OR ("Republica Dominicana") OR ("Ecuador") OR ("El Salvador") OR ("Granada") OR ("Guatemala") OR ("Guyana") OR ("Haití") OR ("Honduras") OR ("Jamaica") OR ("México") OR ("Nicaragua") OR ("Panamá") OR ("Paraguay") OR ("Perú") OR ("Puerto Rico") OR ("San Cristóbal y Nieves ") OR ("Santa Lucía") OR ("San Vicente y las Granadinas ") OR ("Surinam") OR ("Trinidad y Tobago") OR ("Turcas y Caicos ") OR ("Uruguay") OR ("Venezuela") OR ("América Latina") OR ("Latinoamérica") OR ("América del Sur") OR ("Sudamérica") OR ("Suramérica") OR ("América Central") OR ("Centroamérica") OR ("América del Centro") OR ("Caribe"))

Carrillo-Larco RM, *et al. BMJ Open Diab Res Care* 2020; 8:e001169. doi: 10.1136/bmjdrc-2019-001169

Data extraction form (by chapters)

Source of data and participants

	Source of data	Participants									
Study	Source of data	Participant location	Baseline year	End year (cohorts)	Sampling	Inclusion criteria	Exclusion criteria	Outcome prevalence (%)	Outcome incidence (for cohorts)	Baseline mean age	Baseline % men
Gomez-Arbelaez, 2015	Cross-sectional	Health centres	2012		Convenience	Adult subjects >=35 years who attended the general practitioner for any reason at the ambulatory service, express interest in participating, and had laboratory tests on the hospital's database.	People with known diabetes mellitus (type 1 or 2) were not recruited; any acute illness, pregnancy in women, and currently use of metformin or other glucose-modifying prescription drugs.	2.59		58.34	29.53
Bernabe-Ortiz, 2018 - FINDRISC Simplified	Cross-sectional	Community	2016-2017		Random	People aged 30-69 years, full time residents and able to understand procedures and provide informed consent	Pregnant women and people with any physical disability preventing clinical (e.g., anthropometrics) assessment	4.70		48.20	49.70
Bernabe-Ortiz, 2018 - FINDRISC	Cross-sectional	Community	2016-2017		Random	People aged 30-69 years, full time residents and able to understand procedures and provide informed consent	Pregnant women and people with any physical disability preventing clinical (e.g., anthropometrics) assessment	4.70		48.20	49.70
Bernabe-Ortiz, 2018 - LA-FINDRISC	Cross-sectional	Community	2016-2017		Random	People aged 30-69 years, full time residents and able to understand procedures and provide informed consent	Pregnant women and people with any physical disability preventing clinical (e.g., anthropometrics) assessment	4.70		48.20	49.70

Carrillo-Larco RM, *et al. BMJ Open Diab Res Care* 2020; 8:e001169. doi: 10.1136/bmjdrc-2019-001169

Nieto-Martinez, 2019 - LA-FINDRISC	Cross-sectional	Community	2014-2017		Random	Subjects aged 20+ years of age living in the house selected for more than six months.	Current pregnancy, inability to stand or communicate, or refusal to participate in the study by not signing the informed consent.	3.30		39.90	46.97
Nieto-Martinez, 2019 - FINDRISC	Cross-sectional	Community	2014-2017		Random	Subjects aged 20+ years of age living in the house selected for more than six months.	Current pregnancy, inability to stand or communicate, or refusal to participant in the study by not signing the informed consent.	3.30		39.90	46.97
Barengo, 2017 - ColDRISC	Cross-sectional	Health centres	2014-2015		Random	Subjects aged 18-74 and signed informed consent.	Drug treatment for T2DM or previously diagnosed diabetes; pregnancy or breastfeeding; history of cancer; regular use of systemic corticosteroids; haemophilia; inability to stand or communicate; living in area of difficult access.	5.10		47.20	38.00
Barengo, 2017 - modified FINDRISC	Cross-sectional	Health centres	2014-2015		Random	Subjects aged 18-74 and signed informed consent.	Drug treatment for T2DM or previously diagnosed diabetes; pregnancy or breastfeeding; history of cancer; regular use of systemic corticosteroids; haemophilia; inability to stand or communicate; living in area of difficult access.	5.10		47.20	38.00

Carrillo-Larco RM, *et al. BMJ Open Diab Res Care* 2020; 8:e001169. doi: 10.1136/bmjdrc-2019-001169

Outcome

	Outcome					
Study	Outcome	Outcome details	Same outcome definition for all patients?	Blinded outcome	Predictors part of the outcome	Mean follow-up (years) (cohorts)
Gomez-Arbelaez, 2015	Diabetes lab-only	The diagnosis of type 2 diabetes mellitus was established when fasting plasma glucose >= 126 mg/dL, OGTT >=200 mg/dL and/or HbA1c >= 6.5%.	Yes	Yes	No	
Bernabe-Ortiz, 2018 - FINDRISC Simplified	Diabetes lab-only	Individuals who were not aware of having type 2 diabetes mellitus and had fasting glucose ≥126 mg/dL (≥7.0 mmol/L) or 2-hour plasma glucose ≥200 mg/dL (≥11.1 mmol/L)	Yes	Yes	No	
Bernabe-Ortiz, 2018 - FINDRISC	Diabetes lab-only	Individuals who were not aware of having type 2 diabetes mellitus and had fasting glucose ≥126 mg/dL (≥7.0 mmol/L) or 2-hour plasma glucose ≥200 mg/dL (≥11.1 mmol/L)	Yes	Yes	No	
Bernabe-Ortiz, 2018 - LA-FINDRISC	Diabetes lab-only	Individuals who were not aware of having type 2 diabetes mellitus and had fasting glucose ≥126 mg/dL (≥7.0 mmol/L) or 2-hour plasma glucose ≥200 mg/dL (≥11.1 mmol/L)	Yes	Yes	No	
Nieto-Martinez, 2019 - LA-FINDRISC	Diabetes lab-only	Diabetes was defined if the fasting plasma glucose was >=126 mg/dL or if the 2-h OGTT glucose was >=200 mg/dL.	Yes	Yes	No	
Nieto-Martinez, 2019 - FINDRISC	Diabetes lab-only	Diabetes was defined if the fasting plasma glucose was >=126 mg/dL or if the 2-h OGTT glucose was >=200 mg/dL.	Yes	Yes	No	
Barengo, 2017 - ColDRISC	Diabetes lab-only	Individuals who had fasting plasma glucose >= 126 mg/dl or 2-hour plasma glucose >= 200 mg/dl were classified as having T2DM.	Yes	Yes	No	
Barengo, 2017 - modified FINDRISC	Diabetes lab-only	Individuals who had fasting plasma glucose >= 126 mg/dl or 2-hour plasma glucose >= 200 mg/dl were classified as having T2DM.	Yes	Yes	No	

Carrillo-Larco RM, *et al. BMJ Open Diab Res Care* 2020; 8:e001169. doi: 10.1136/bmjdrc-2019-001169

Candidate predictors

	Candidate Predictors					
Study	Number of candidate predictors	Number of predictors in the final model	Predictors timing	List of predictors in the final model	Predictors definition	Predictors ascertainment
Gomez-Arbelaez, 2015		13	Baseline	Age, body mass index, waist circumference, current antihypertensive medication, frequency of fruit/vegetable consumption, physical activity, personal history of high blood glucose, and family history of type 2 diabetes mellitus.	Age (<45, 45-54, 55-64, >64) , body mass index/BMI (<25, 25-30, >30); waist circumference/WC (men <90 and women <80, men 90-98 and women 80-88, men >98 and women >88); current antihypertensive medication (with or without); frequency of fruit and vegetable consumption (daily or not); physical activity (at least 30 minutes per day); personal history of high blood glucose (yes or no); and family history of DM2 (none, grandparents, parents).	People were asked to complete a modified version of the FINDRISC score. General practitioners performed anthropometric measurements. Laboratory tests were collected directly from the hospital's database; only those tests taken within the two months previous or after the survey were valid for the study.
Bernabe-Ortiz, 2018 - FINDRISC Simplified	12	5	Baseline	Waist circumference; blood pressure medication; history of high blood glucose levels; family history of type 2 diabetes mellitus	Waist circumference (vs F<80cm/M<94cm), F>=80<88cm/M>=94<102, F>=88cm/M>=102. Blood pressure medication (vs No) Yes. History of high blood glucose levels (vs No) Yes. Family history of type 2 diabetes mellitus (vs No) parents, brother, sister or own child.	Questionnaires and clinical evaluation (anthropometrics)
Bernabe-Ortiz, 2018 - FINDRISC		13	Baseline	Age; body mass index; waist circumference; physical activity; fruits and vegetable intake; medication for hypertension; history of high glucose levels; diabetes in relatives	Age [<45 (ref), 45-54, 55-64, ≥65]; body mass index [<25 (ref), 25.-29.99, ≥30]; waist circumference [men <94 & women <80, men 94-102 & women 80-88, men >102 & women >88]; physical activity at least 30 min/day [yes (ref), no]; fruits and vegetables intake [every day (ref), not every day]; regular medication for hypertension [no (ref), yes]; history of high glucose levels [no (ref), yes]; diabetes in relatives [no (ref), yes in grandparents/cousins/uncle/aunt, yes in parents/siblings/offspring]	Questionnaires and clinical evaluation (anthropometrics)
Bernabe-Ortiz, 2018 - LA-FINDRISC		12	Baseline	Age; body mass index; waist circumference; physical activity; fruits and vegetable intake; medication for hypertension; history of high glucose levels; diabetes in relatives	Age [<45 (ref), 45-54, 55-64, ≥65]; body mass index [<25 (ref), 25.-29.99, ≥30]; waist circumference [men <94 & women <80 (ref), men 94-102 & women 80-88]; physical activity at least 30 min/day [yes (ref), no]; Fruits and vegetables intake [every day (ref), not every day]; regular medication for hypertension [no (ref), yes]; history of high glucose levels [no (ref), yes]; diabetes in relatives [no (ref), yes in grandparents/cousins/uncle/aunt, yes in parents/siblings/offspring]	Questionnaires and clinical evaluation (anthropometrics)
Nieto-Martinez		12	Baseline	Age; body mass index; waist circumference; physical activity; fruits and vegetable intake;	Age [<45 (ref), 45-54, 55-64, ≥65]; body mass index [<25 (ref), 25.-29.99, ≥30]; waist circumference [men <94 & women <80 (ref), men	Questionnaires and clinical evaluation (anthropometrics)

, 2019 - LA-FINDRISC				medication for hypertension; history of high glucose levels; diabetes in relatives	>=94 & women >=80]; physical activity at least 30 min/day 5 times a week [yes (ref), no]; fruits and vegetables intake [every day (ref), not every day]; regular medication for hypertension [no (ref), yes]; history of high glucose levels [no (ref), yes]; diabetes in relatives [no, (ref), yes in grandparents/cousins/uncle/aunt, yes in parents/siblings/offspring]	
Nieto-Martinez , 2019 - FINDRISC		13	Baseline	Age; body mass index; waist circumference; physical activity; fruits and vegetable intake; medication for hypertension; history of high glucose levels; diabetes in relatives	Age [<45 (ref), 45-54, 55-64, ≥65]; body mass index [<25 (ref), 25.-29.99, ≥30]; waist circumference [men <94 & women <80, men 94-102 & women 80-88, men >102 & women >88]; physical activity at least 30 min/day [yes (ref), no]; fruits and vegetables intake [every day (ref), not every day]; regular medication for hypertension [no (ref), yes]; history of high glucose levels [no (ref), yes]; diabetes in relatives [no, (ref), yes in grandparents/cousins/uncle/aunt, yes in parents/siblings/offspring]	Questionnaires and clinical evaluation (anthropometrics)
Barengo, 2017 - ColDRISC	9	6	Baseline	Age; waist circumference; use of antihypertensive medication; family history of diabetes mellitus.	Age [<45 (ref); 45-54; 55-64; 64+]; waist circumference [94+ in men and 90+ in women, vs otherwise]; use of blood pressure medication (yes/no (ref)); family history of diabetes mellitus [biological father, mother or sibling].	Questionnaire based on the FINDRISC and the WHO STEPS approach and IPAQ questionnaire. Waist circumference was measured at the approximate midpoint between the lower margin of the last palpable rib and the top of the celiac crest.
Barengo, 2017 - modified FINDRISC		12	Baseline	Age; body mass index; waist circumference; physical activity; fruits and vegetable intake; medication for hypertension; history of high glucose levels; diabetes in relatives	Age [<45 (ref), 45-54, 55-64, ≥65]; body mass index [<25 (ref), 25.-29.99, ≥30]; waist circumference [men <94 & women <90 (ref), men >=94 & women >=90]; physical activity at least 30 min/day [yes (ref), no]; Fruits and vegetables intake [every day (ref), not every day]; regular medication for hypertension [no (ref), yes]; history of high glucose levels [no (ref), yes]; diabetes in relatives [no (ref), yes in grandparents/cousins/uncle/aunt, yes in parents/siblings/offspring]	Questionnaires and clinical evaluation (anthropometrics)

Carrillo-Larco RM, *et al. BMJ Open Diab Res Care* 2020; 8:e001169. doi: 10.1136/bmjdrc-2019-001169

Sample size and missing data

	Sample Size			Missing Data		
Study	Baseline sample size	Number of outcome events	Total outcome events per candidate predictors	Missing data	Number of participants with missing data	Missing data per candidate predictors
Gomez-Arbelaez, 2015	772	20		NI		
Bernabe-Ortiz, 2018 - FINDRISC Simplified	1609	71	5.92	Complete-case	3	0.25
Bernabe-Ortiz, 2018 - FINDRISC	1609	71		Complete-case	3	
Bernabe-Ortiz, 2018 - LA-FINDRISC	1609	71		Complete-case	3	
Nieto-Martinez, 2019 - LA-FINDRISC	3061	101		Complete-case	38	
Nieto-Martinez, 2019 - FINDRISC	3061	101		Complete-case	38	
Barengo, 2017 - ColDRISC	2060	105	11.67	Complete-case	553	61.44
Barengo, 2017 - modified FINDRISC	2060	105		Complete-case	553	

Carrillo-Larco RM, *et al. BMJ Open Diab Res Care* 2020; 8:e001169. doi: 10.1136/bmjdrc-2019-001169

Model development

	Model Development					
Study	Regression method	Were the model assumptions verified?	Predictors selection	If the prediction model was a replication, which was the original model?	If there were pre-selection, describe the method	Was a shrinkage method used?
Gomez-Arbelaez, 2015						
Bernabe-Ortiz, 2018 - FINDRISC Simplified	Logistic	NI	Pre-selection	FINDRISC	Stepwise backward elimination	No
Bernabe-Ortiz, 2018 - FINDRISC						
Bernabe-Ortiz, 2018 - LA-FINDRISC						
Nieto-Martinez, 2019 - LA-FINDRISC						
Nieto-Martinez, 2019 - FINDRISC						
Barengo, 2017 - ColDRISC	Logistic	NI	Pre-selection	FINDRISC	Univariate selection	No
Barengo, 2017 - modified FINDRISC						

Carrillo-Larco RM, *et al. BMJ Open Diab Res Care* 2020; 8:e001169. doi: 10.1136/bmjdrc-2019-001169

Model performance

	Model Performance				
Study	Calibration	Discrimination (%)	Classification measures	Cut-off point	For replication studies, was the cut-off the same?
Gomez-Arbelaez, 2015		74.77 [95% CI: 57.22-92.32] (men) and 71.75 [95% CI: 58.68-84.81] (women)	At a cut-off of >=14. Men: sensitivity = 66.7; specificity = 75.2; PPV = 6.8; NPV = 98.8; Youden's index = 0.419. Women: sensitivity = 71.4; specificity = 62.6; PPV = 4.8; NPV = 98.8; Youden's index = 0.340.	14	No
Bernabe-Ortiz, 2018 - FINDRISC Simplified		71.10	At a cut-off 3. Sensitivity=0.859; specificity=0.467; positive predictive value=0.074; negative predictive value=0.985; likelihood ratio positive=1.6; likelihood ratio negative=0.3; diagnostic odd ratio=5.3	3	No
Bernabe-Ortiz, 2018 - FINDRISC		69.00	At a cut-off 11. Sensitivity=0.690; specificity=0.668; positive predictive value=0.094; negative predictive value=0.978; likelihood ratio positive=2.1; likelihood ratio negative=0.5; diagnostic odd ratio=4.5	11	No
Bernabe-Ortiz, 2018 - LA-FINDRISC		68.00	At a cut-off 10. Sensitivity=0.704; specificity=0.591; positive predictive value=0.079; negative predictive value=0.970; likelihood ratio positive=1.7; likelihood ratio negative=0.5; diagnostic odd ratio=3.4	10	No
Nieto-Martinez, 2019 - LA-FINDRISC		72.2 [95%CI: 66.8–77.5] (men) and 72.40 [95% CI: 63.9–81.0] (women)	For men at a cut-off 9. Sensitivity = 72.2; specificity = 62.2; + likelihood ratio = 1.91. For women at a cut-off 10. Sensitivity = 71.4; specificity = 65.4; + likelihood ratio = 2.06.	9 (men) and 10 (women)	Yes
Nieto-Martinez, 2019 - FINDRISC		72.90 [95% CI: 67.6–78.1] (men) and 73.20 [95% CI: 64.8–81.6] (women)			
Barengo, 2017 - ColDRISC		74 (95% CI: 70-79)	At a cut-off 4. sensitivity=0.73; specificity=0.67; positive predictive value=0.106; negative predictive value=0.979	4	No
Barengo, 2017 - modified FINDRISC		73 (95% CI: 69-78)	At a cut-off 10. sensitivity=0.72; specificity=0.60; positive predictive value=0.084; negative predictive value=0.984	10	No

Results

	Results					
Study	Was a simplified model presented?	Were the coefficients of the regression model presented?	Report the coefficients	Report the intercept	Was the baseline risk presented?	Were there alternative results presentation?
Gomez-Arbelaez, 2015						
Bernabe-Ortiz, 2018 - FINDRISC Simplified	Yes	Yes	Waist circumference F>=80<88/M>=94<102cm [1.03 (SE=0.45)]; F>=88/M>=102 [1.30 (SE=0.42)]. Blood pressure medication Yes [0.98 (SE=0.33)]. History of high blood glucose levels Yes [1.19 (SE=0.42)]. Family history of type 2 diabetes mellitus Yes [0.61 (SE=0.25)].	No		Yes
Bernabe-Ortiz, 2018 - FINDRISC						
Bernabe-Ortiz, 2018 - LA-FINDRISC						
Nieto-Martinez, 2019 - LA-FINDRISC						
Nieto-Martinez, 2019 - FINDRISC						
Barengo, 2017 - ColDRISC	No	No		No		No
Barengo, 2017 - modified FINDRISC						

Discussion

	Discussion		
Study	Interpretation of the results	Comparison with other studies in LAC	Generalizability
Gomez-Arbelaez, 2015	Confirmatory	Yes	Non-generalizability
Bernabe-Ortiz, 2018 - FINDRISC Simplified	Exploratory	Yes	Non-generalizability
Bernabe-Ortiz, 2018 - FINDRISC	Exploratory	Yes	Non-generalizability
Bernabe-Ortiz, 2018 - LA-FINDRISC	Exploratory	Yes	Non-generalizability
Nieto-Martinez, 2019 - LA-FINDRISC	Confirmatory	Yes	NI
Nieto-Martinez, 2019 - FINDRISC	Confirmatory	No	NI
Barengo, 2017 - ColDRISC	Confirmatory	Yes	Non-generalizability
Barengo, 2017 - modified FINDRISC	Confirmatory	Yes	Non-generalizability

Carrillo-Larco RM, *et al. BMJ Open Diab Res Care* 2020; 8:e001169. doi: 10.1136/bmjdrc-2019-001169

Risk of Bias (RoB)

Study	Participants		Predictors		
	Were appropriate data sources used, e.g., cohort, RCT, or nested case–control study data?	Were all inclusions and exclusions of participants appropriate?	Were predictors defined and assessed in a similar way for all participants?	Were predictor assessments made without knowledge of outcome data?	Are all predictors available at the time the model is intended to be used?
Barengo, 2017 (ColDRISC derivation)	Y	Y	Y	Y	Y
Barengo, 2017 (LA-FINDRISC validation)	Y	Y	Y	Y	Y
Bernabe-Ortiz, 2018 - FINDRISC Simplified (derivation)	Y	Y	Y	Y	Y
Bernabe-Ortiz, 2018 - FINDRISC (validation)	Y	Y	Y	Y	Y
Bernabe-Ortiz, 2018 - LA-FINDRISC (validation)	Y	Y	Y	Y	Y
Bernabe-Ortiz, 2018 - Peruvian Risk Score (validation)	Y	Y	Y	Y	Y
Gomez-Arbelaez, 2015 (validation)	Y	Y	Y	PY	Y
Nieto-Martínez, 2019 LA-FINDRISC (validation)	Y	Y	Y	PY	Y
Nieto-Martínez, 2019 - FINDRISC (validation)	Y	Y	Y	PY	Y

Carrillo-Larco RM, *et al. BMJ Open Diab Res Care* 2020; 8:e001169. doi: 10.1136/bmjdrc-2019-001169

Study	Outcome					
	**Was the outcome determined appropriately?**	**Was a prespecified or standard outcome definition used?**	**Were predictors excluded from the outcome definition?**	**Was the outcome defined and determined in a similar way for all participants?**	**Was the outcome determined without knowledge of predictor information?**	**Was the time interval between predictor assessment and outcome determination appropriate?**
Barengo, 2017 (ColDRISC derivation)	Y	Y	Y	Y	Y	Y
Barengo, 2017 (LA-FINDRISC validation)	Y	Y	Y	Y	Y	Y
Bernabe-Ortiz, 2018 - FINDRISC Simplified (derivation)	Y	Y	Y	Y	Y	Y
Bernabe-Ortiz, 2018 - FINDRISC (validation)	Y	Y	Y	Y	Y	Y
Bernabe-Ortiz, 2018 - LA-FINDRISC (validation)	Y	Y	Y	Y	Y	Y
Bernabe-Ortiz, 2018 - Peruvian Risk Score (validation)	Y	Y	Y	Y	Y	Y
Gomez-Arbelaez, 2015 (validation)	Y	Y	Y	Y	PY	N
Nieto-Martínez, 2019 LA-FINDRISC (validation)	Y	Y	Y	Y	PY	Y
Nieto-Martínez, 2019 - FINDRISC (validation)	Y	Y	Y	Y	PY	Y

Study	Analysis								
	Were there a reasonable number of participants with the outcome?	Were continuous and categorical predictors handled appropriately?	Were all enrolled participants included in the analysis?	Were participants with missing data handled appropriately?	Was selection of predictors based on univariable analysis avoided? [development studies only]	Were complexities in the data (e.g., censoring, competing risks, sampling of control participants) accounted for appropriately?	Were relevant model performance measures evaluated appropriately?	Were model overfitting and optimism in model performance accounted for? [development studies only]	Do predictors and their assigned weights in the final model correspond to the results from the reported multivariable analysis? [development studies only]
Barengo, 2017 (ColDRISC derivation)	PY	Y	N	N	N	PY	N	N	NI
Barengo, 2017 (LA-FINDRISC validation)	Y	Y	N	N	n/a	PY	N	n/a	n/a
Bernabe-Ortiz, 2018 - FINDRISC Simplified (derivation)	N	Y	N	PN	N	PY	N	N	Y
Bernabe-Ortiz, 2018 - FINDRISC (validation)	PN	Y	N	PN	n/a	PY	N	n/a	n/a
Bernabe-Ortiz, 2018 - LA-FINDRISC (validation)	PN	Y	N	PN	n/a	PY	N	n/a	n/a
Bernabe-Ortiz, 2018 - Peruvian Risk Score (validation)	PN	Y	N	PN	n/a	PY	N	n/a	n/a
Gomez-Arbelaez, 2015 (validation)	N	N	N	Y	n/a	PY	N	n/a	n/a
Nieto-Martínez, 2019 LA-FINDRISC (validation)	Y	Y	Y	PN	n/a	PY	N	n/a	n/a
Nieto-Martínez, 2019 - FINDRISC (validation)	Y	Y	Y	PN	n/a	PY	N	n/a	n/a

Applicability

Study	Participants	Predictors	Outcome
Barengo, 2017 (ColDRISC derivation)	Low	Low	Low
Barengo, 2017 (LA-FINDRISC validation)	Low	Low	Low
Bernabe-Ortiz, 2018 - FINDRISC Simplified (derivation)	Low	Low	Low
Bernabe-Ortiz, 2018 - FINDRISC (validation)	Low	Low	Low
Bernabe-Ortiz, 2018 - LA-FINDRISC (validation)	Low	Low	Low
Bernabe-Ortiz, 2018 - Peruvian Risk Score (validation)	Low	Low	Low
Gomez-Arbelaez, 2015 (validation)	Low	Low	Low
Nieto-Martínez, 2019 LA-FINDRISC (validation)	Low	Low	Low
Nieto-Martínez, 2019 - FINDRISC (validation)	Low	Low	Low

Carrillo-Larco RM, *et al. BMJ Open Diab Res Care* 2020; 8:e001169. doi: 10.1136/bmjdrc-2019-001169

PRISMA Checklist

Section/topic	#	Checklist item	Reported on page #
**TITLE**			
Title	1	Identify the report as a systematic review, meta-analysis, or both.	01
**ABSTRACT**			
Structured summary	2	Provide a structured summary including, as applicable: background; objectives; data sources; study eligibility criteria, participants, and interventions; study appraisal and synthesis methods; results; limitations; conclusions and implications of key findings; systematic review registration number.	03
**INTRODUCTION**			
Rationale	3	Describe the rationale for the review in the context of what is already known.	04
Objectives	4	Provide an explicit statement of questions being addressed with reference to participants, interventions, comparisons, outcomes, and study design (PICOS).	04
**METHODS**			
Protocol and registration	5	Indicate if a review protocol exists, if and where it can be accessed (e.g., Web address), and, if available, provide registration information including registration number.	05
Eligibility criteria	6	Specify study characteristics (e.g., PICOS, length of follow-up) and report characteristics (e.g., years considered, language, publication status) used as criteria for eligibility, giving rationale.	05
Information sources	7	Describe all information sources (e.g., databases with dates of coverage, contact with study authors to identify additional studies) in the search and date last searched.	05
Search	8	Present full electronic search strategy for at least one database, including any limits used, such that it could be repeated.	05
Study selection	9	State the process for selecting studies (i.e., screening, eligibility, included in systematic review, and, if applicable, included in the meta-analysis).	05-06
Data collection process	10	Describe method of data extraction from reports (e.g., piloted forms, independently, in duplicate) and any processes for obtaining and confirming data from investigators.	06

Carrillo-Larco RM, *et al. BMJ Open Diab Res Care* 2020; 8:e001169. doi: 10.1136/bmjdrc-2019-001169

Data items	11	List and define all variables for which data were sought (e.g., PICOS, funding sources) and any assumptions and simplifications made.	06
Risk of bias in individual studies	12	Describe methods used for assessing risk of bias of individual studies (including specification of whether this was done at the study or outcome level), and how this information is to be used in any data synthesis.	06
Summary measures	13	State the principal summary measures (e.g., risk ratio, difference in means).	NA
Synthesis of results	14	Describe the methods of handling data and combining results of studies, if done, including measures of consistency (e.g., I^2) for each meta-analysis.	06
Risk of bias across studies	15	Specify any assessment of risk of bias that may affect the cumulative evidence (e.g., publication bias, selective reporting within studies).	NA
Additional analyses	16	Describe methods of additional analyses (e.g., sensitivity or subgroup analyses, meta-regression), if done, indicating which were pre-specified.	NA
**RESULTS**			
Study selection	17	Give numbers of studies screened, assessed for eligibility, and included in the review, with reasons for exclusions at each stage, ideally with a flow diagram.	08
Study characteristics	18	For each study, present characteristics for which data were extracted (e.g., study size, PICOS, follow-up period) and provide the citations.	08
Risk of bias within studies	19	Present data on risk of bias of each study and, if available, any outcome level assessment (see item 12).	09
Results of individual studies	20	For all outcomes considered (benefits or harms), present, for each study: (a) simple summary data for each intervention group (b) effect estimates and confidence intervals, ideally with a forest plot.	08-09
Synthesis of results	21	Present results of each meta-analysis done, including confidence intervals and measures of consistency.	08-09
Risk of bias across studies	22	Present results of any assessment of risk of bias across studies (see Item 15).	NA
Additional analysis	23	Give results of additional analyses, if done (e.g., sensitivity or subgroup analyses, meta-regression [see Item 16]).	NA
**DISCUSSION**			
Summary of evidence	24	Summarize the main findings including the strength of evidence for each main outcome; consider their relevance to key groups (e.g., healthcare providers, users, and policy makers).	10
Limitations	25	Discuss limitations at study and outcome level (e.g., risk of bias), and at review-level (e.g., incomplete retrieval of identified research, reporting bias).	10

Conclusions	26	Provide a general interpretation of the results in the context of other evidence, and implications for future research.	11
**FUNDING**			
Funding	27	Describe sources of funding for the systematic review and other support (e.g., supply of data); role of funders for the systematic review.	02

From: Moher D, Liberati A, Tetzlaff J, Altman DG, The PRISMA Group (2009). Preferred Reporting Items for Systematic Reviews and Meta-Analyses: The PRISMA Statement. PLoS Med 6(7): e1000097. doi:10.1371/journal.pmed1000097
